# Safety and effectiveness of the anti‐amyloid monoclonal antibody (mAb) drug lecanemab for early Alzheimer's disease: The pharmacovigilance perspective

**DOI:** 10.1002/bcp.70021

**Published:** 2025-03-06

**Authors:** Amy Bobbins, Miranda Davies, Elizabeth Lynn, Debabrata Roy, Alison Yeomans, Saad A. W. Shakir

**Affiliations:** ^1^ Drug Safety Research Unit Southampton UK; ^2^ School of Pharmacy and Biomedical Sciences University of Portsmouth Portsmouth UK

**Keywords:** Alzheimer's disease, anti‐amyloid monoclonal antibodies, benefit–risk profile, dementia, lecanemab

## Abstract

The development of humanized IgG1 anti‐amyloid monoclonal antibodies, such as lecanemab, provides a promising novel treatment pathway with potential disease‐modifying effects for patients with early Alzheimer's disease (AD). Lecanemab, which gained marketing approval by the United States Food and Drug Administration (US FDA) in July 2023, has since been approved in multiple countries, including the United Kingdom (UK). The decision by the UK's Medicines and Healthcare products Regulatory Agency (MHRA) to approve lecanemab in August 2024 followed similar regulatory decisions in the US and Japan. However, at the time of approval, the decision contrasted with that of the European Medicine Agency (EMA) in July 2024. Subsequently, the EMA recommended the marketing approval of lecanemab in November 2024 following a re‐examination of further data submitted by the Marketing Authorisation Holder. The UK's National Institute for Health and Care Excellence (NICE) has not recommended lecanemab for use in early AD amid concerns, including treatment cost and the translation of efficacy outcomes into clinically meaningful improvement. The risks of serious adverse events (SAEs), including amyloid‐related imaging abnormalities (ARIA), have also emerged from clinical trial data with a concern for the potential for rare, life‐threatening events. This narrative review discusses the requirement for a robust method of monitoring the safety and effectiveness of lecanemab in the real‐world clinical setting considering recent regulatory decisions. Additionally, the need to evaluate proposed risk minimization measures (RMMs) is discussed considering the resource constraints of healthcare systems, such as those faced by the UK's National Health Service (NHS).

## Graphical abstract

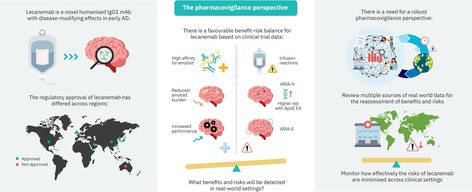

## 1 INTRODUCTION

On 22 August 2024, the United Kingdom's Medicines and Healthcare products Regulatory Agency (MHRA) approved the humanized IgG1 anti‐amyloid monoclonal antibody (mAb) lecanemab for the treatment of mild cognitive impairment (MCI) and mild dementia due to Alzheimer's disease (AD) in patients who are apolipoprotein E ε4 (ApoE ε4) heterozygotes or non‐carriers. Administered at a dose of 10 mg/kg every 2 weeks as an intravenous (IV) infusion, lecanemab binds to, neutralizes and eliminates amyloid‐beta (Aβ) aggregates (protofibrils) suggested to contribute to the neurodegenerative process in AD.^1^ Lecanemab first gained traditional marketing approval by the US Food and Drug Administration (FDA) in July 2023 following conditional approval via the Accelerated Approval Pathway in early 2023.^2^

As the treatment of AD remains an unmet medical need, lecanemab first received Accelerated Approval from the US FDA following positive results from a Phase 3 global confirmatory Clarity AD clinical trial (NCT03887455). This Phase 3 trial was a multicentre randomized, double‐blind, parallel‐group study of 1795 patients with AD investigating the efficacy of lecanemab compared to placebo in early AD dementia (*n* = 1795).^1^ At the completion of the study at 18 months, 729 patients were in the lecanemab arm and 757 in the placebo arm (*n* = 1486). The mean age of participants was 71 years and the trial included data from 235 sites globally.^3^ Consideration of the benefits and risks arising from the use of lecanemab from the Clarity AD trial data provides an indicator of the initial benefit–risk balance of the product. In this narrative review, we will discuss the need for ongoing monitoring and assessment of the safety and effectiveness of lecanemab in a real‐world setting based on the initial benefit–risk profile of the drug and in the context of recent regulatory decisions. Additionally, we consider the feasibility of providing the proposed risk minimization measures (RMMs) to reduce the risk of adverse events (AEs) in healthcare systems, particularly those with limited resource availability.

## 2 A COMPLEX GLOBAL REGULATORY LANDSCAPE

**FIGURE 1:**
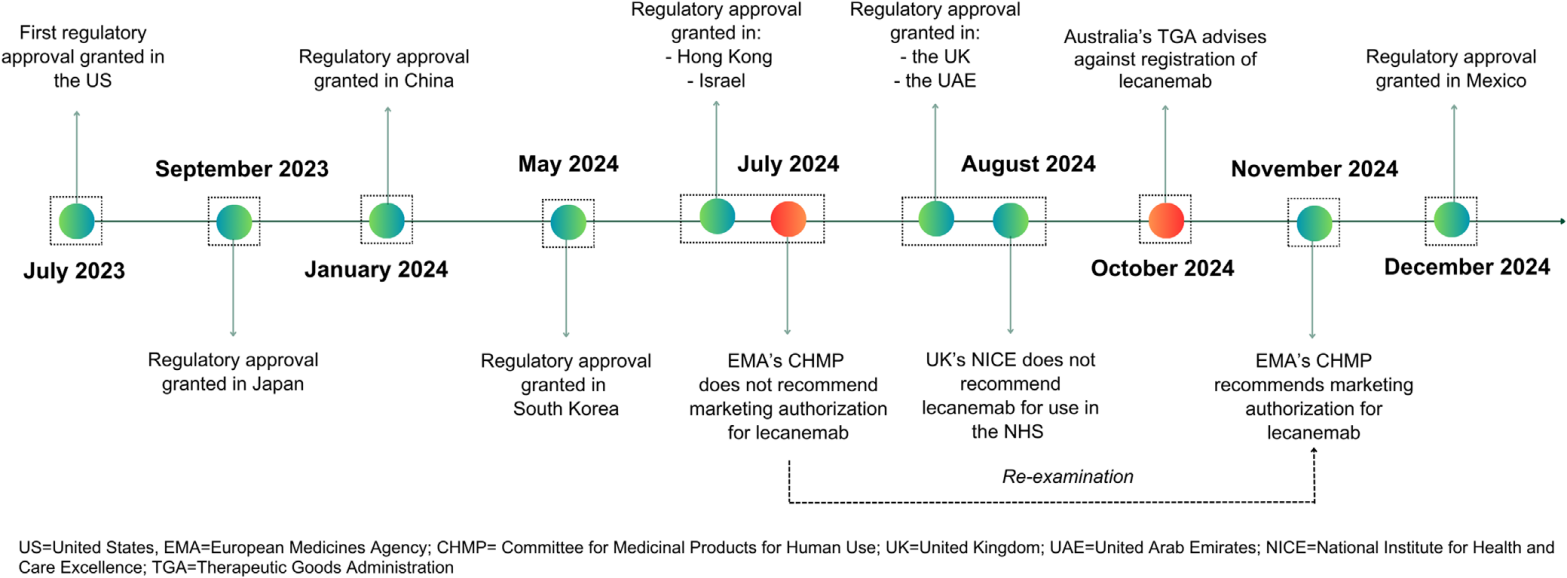
A timeline of major regulatory decisions for lecanemab.

The decision by the UK's MHRA to grant marketing approval for lecanemab in August 2024 followed similar regulatory decisions in the US, Japan, China, South Korea, Hong Kong, Israel and the United Arab Emirates (UAE). The MHRA had approved lecanemab for use in the UK in contrast to the decision made in July 2024 a month prior by the European Medicine Agency (EMA). The EMA's Committee for Medicinal Products for Human Use (CHMP) initially refused the marketing approval of lecanemab in July 2024 determining that the observed benefits of treatment did not outweigh the risk of serious adverse events (SAEs) associated with the use of the drug in early disease.^4^ However, following a re‐examination of its initial opinion and further submission of data from the Marketing Authorization Holder (MAH), the EMA recommended the marketing approval of lecanemab in November 2024 with use restricted to patients with only one or no copy of ApoE ε4.^5^ In recent months, lecanemab has been refused marketing approval in Australia by the Therapeutic Goods Authority (TGA) (October 2024) but approved for use in Mexico by Mexico's Federal Commission for the Protection Against Sanitary Risk (December 2024) (Figure 1).^6^, ^7^

The availability and access to lecanemab in the UK is limited as the UK's National Institute for Health and Care Excellence (NICE) has not recommended lecanemab for routine use in the National Health Service (NHS).^8^ Despite the lack of available disease‐modifying treatment options for AD, NICE considered there to be high levels of uncertainty regarding the long‐term effectiveness of lecanemab and required more evidence regarding its cost‐effectiveness.^3^ Based on this non‐recommendation, it is likely that the use of this product outside of clinical trial settings will initially be restricted to use in private sector healthcare settings in the UK. Similarly, it is envisaged that lecanemab will be used in the private sector in other countries globally, including the US and China.^9^, ^10^ This has implications for the access to and availability of this product across global settings, with utilization likely to be representative of a demographic of patients who can afford the product privately. For example, upon launch in the US, the projected annual treatment cost was $26 500 per patient (January 2023). This high annual cost introduces uncertainty around the long‐term clinical use of lecanemab and the economic value of lecanemab as an appropriate treatment option for patients with early AD.^10^

## 3 A NOVEL PRODUCT WITH COMPLEX BENEFITS AND RISKS

Consideration of the benefits and risks arising from the use of lecanemab in clinical trials provides an indicator of the initial benefit–risk balance of the product. However, as with all medicinal products, there is a need for ongoing benefit–risk monitoring in the post‐authorization phase due to the acknowledged limitations of clinical trials.^11^, ^12^, ^13^

### 3.1 Benefits

Lecanemab has high selectivity for soluble aggregated species of Aβ with moderate selectivity for the most toxic pathologic amyloid species, fibrillar amyloid.^1^ Lecanemab has shown a reduction in mean amyloid burden in early AD following treatment at 18 months when compared to placebo.^1^, ^14^, ^15^ In the Phase 3 confirmatory clinical trial, the primary outcome was a change in the Clinical Dementia Rating‐Sum of Boxes (CDR‐SB) score with an increase in this score indicating a worsening of dementia severity and progression. Whilst an increase in the score was observed in both the lecanemab and the placebo groups, the lecanemab group increased by an adjusted mean change of 1.213 compared with an adjusted mean increase of 1.663 for placebo. The score indicates a measure of cognitive and functional performance across six domains including memory, orientation, judgement and problem‐solving. This indicated an adjusted mean difference between lecanemab and placebo arms of −0.451 (−27.1%, *P* = 0.00005) in the intention‐to‐treat full analysis set.^3^

However, there have been concerns raised by clinicians regarding the translation of these statistically significant outcomes of efficacy into clinically significant benefits to the cognitive function of patients with early AD.^16^, ^17^ When pooling data from clinical trials of three anti‐amyloid mAb products with a demonstrated high clearance of brain amyloid (aducanumab, lecanemab and donanemab), a statistically significant difference in CDR‐SB scores was observed, although this translated only to a slightly positive clinical benefit after 18 months.^11^, ^18^, ^19^ Although the CDR score is a widely used metric accepted by regulators as a suitable primary endpoint in randomized control trials (RCTs), there is still debate around the interpretation of the clinical meaningfulness of a statistically significant change in this score on clinical outcomes.^17^

The threshold values indicative of a clinically meaningful decline in disease progression are considered to be a change in CDR‐SB ranging from 1–2 points (a change of 0.98 for patients with MCI and a change of 1.63 for patients with mild AD).^3^, ^20^ However, the UK's NICE notes that the actual treatment effect observed with lecanemab use in clinical trials was smaller than both these values. The Royal College of Psychiatrists and the Association of British Neurologists considered this observed treatment effect of lecanemab to be clinically meaningful to people with AD, with a slowing of the disease by 4–6 months. Clinical experts emphasized the need to consider the highly heterogeneous nature of AD with varied slowing of the disease expected across patients.^3^ Similarly, the European Union‐North American Clinical Trials in Alzheimer's Disease Task Force (EU/US CTAD Task Force) advise shifting the focus to observing “Meaningful Within‐Person Change” (MWPC) as a more clinically meaningful impact measure on patient's disease progression.^17^ Importantly, any measure of clinical meaningfulness derived from the analysis of efficacy in RCTs is not directly translatable to that observed in the real world; thus there is a clear need to monitor effectiveness data in real‐world clinical settings, particularly over longer term duration of treatments beyond 18 months.^17^

### 3.2 Risks

In the Phase 3 confirmatory Clarity AD clinical trial, the overall incidence of AEs was similar across the two study arms. The most common AEs in the lecanemab arm were infusion‐related reactions (26.4% *vs*. 7.4% with placebo), amyloid‐related imaging abnormalities (ARIA) with cerebral microhaemorrhages, cerebral macrohaemorrhages or superficial siderosis as ARIA with hemosiderin deposition (ARIA‐H) (17.3% *vs*. 9.0% with placebo) and ARIA with oedema (ARIA‐E) (12.6% *vs*. 1.7% with placebo), headache (11.1% *vs*. 8.1% with placebo), and falls (10.4% *vs*. 9.6% with placebo). SAEs occurred in 14% of participants in the lecanemab arm compared to 11.3% with placebo. The most common SAEs were infusion‐related reactions (1.2% *vs*. 0% with placebo), ARIA‐E (0.8% *vs*. 0% with placebo), atrial fibrillation (0.7% *vs*. 0.3% with placebo), syncope (0.7% *vs*. 0.1% with placebo) and angina pectoris (0.7% *vs*. 0% with placebo). In the lecanemab arm, 6.9% discontinued lecanemab due to AEs *vs*. 2.9% of those in the placebo arm.^1^

ARIA have been noted as a boxed warning in US product information for lecanemab as although usually asymptomatic, ARIA may present with a rare incidence of serious and life‐threatening events, including seizure and status epilepticus.^21^ ARIAs, treatment‐emergent magnetic resonance imaging (MRI) abnormalities, were first observed in the clinical trials for bapineuzumab in 2009 and have since been associated with anti‐amyloid mAbs more broadly.^22^, ^23^ A synergistic pathophysiological mechanism has been proposed for ARIA following the use of anti‐amyloid mAbs, involving a gradual loss in cerebral vascular integrity and impaired perivascular clearance.^24^, ^25^, ^26^ Pre‐existing Aβ plaques are broken down into Aβ aggregates with the immune response increasing vascular permeability.^25^, ^26^ As Aβ_40_ is the predominant amyloid species in vascular walls and Aβ_42_ deposits are the major species in parenchymal plaques, mAbs may bind to these Aβ species in brain parenchyma and vasculature, reducing their integrity.^26^, ^27^ Additionally, in AD, the perivascular clearance pathways are compromised which promotes amyloid vascular accumulation. The extravasation of blood products through permeated vessel walls results, including proteinaceous fluid (ARIA‐E) and erythrocytes (ARIA‐H).^25^ ARIA‐H and ARIA‐E often co‐occur due to the presence and accumulation of microhaemorrhages over time in areas where ARIA‐E is resolving or has resolved.^24^, ^26^, ^27^

In clinical trial data, the risk of ARIA with lecanemab was increased in patients who were ApoE ε4 homozygotes compared to patients who were ApoE ε4 heterozygotes and ApoE ε4 non‐carriers.^1^, ^14^, ^15^ In the Clarity AD trial, 620 out of 898 patients were ApoE ε4 carriers. The highest frequency of events of ARIA‐E and ARIA‐H were observed in ApoE ε4 homozygotes, with 46 out of 141 homozygous patients experiencing events of ARIA‐E (32.6%) and 52 out of 479 experiencing events of ARIA‐H (10.9%), compared with 9 of 478 patients (1.9%) with ARIA‐E and 5 out of 133 (3.8%) with ARIA‐H in the placebo arm^1^ (Table 1).

**TABLE 1:** Events of ARIA‐E and ARIA‐H for lecanemab and placebo according to ApoE ε4 status in the Clarity AD trial.^1^

ARIA‐E no./total no. (%)			ARIA‐H no./total no. (%)		
ApoE ε4 status	Lecanemab (N = 898)	Placebo (N = 897)	ApoE ε4 status	Lecanemab (N = 898)	Placebo (N = 897)
**Non‐carrier**	15/278 (5.4)	1/286 (0.3)	**Non‐carrier**	33/278 (11.9)	12/286 (4.2)
**Carrier**	98/620 (15.8)	14/611 (2.3)	**Carrier**	122/620 (19.7)	69/611 (11.3)
Heterozygote	52/479 (10.9)	9/478 (1.9)	Heterozygote	67/479 (14.0)	41/478 (8.6)
Homozygote	46/141 (32.6)	5/133 (3.8)	Homozygote	55/141 (39.0)	28/133 (21.1)

Abbreviations: ARIA, amyloid‐related imaging abnormalities; ARIA‐E, ARIA with oedema or effusions; ARIA‐H, ARIA with hemosiderin deposits; ApoE ε4, apolipoprotein E ε4.

There is strong evidence to suggest that the ApoE ε4 genotype is one of the main risk factors for the development of ARIA‐E and ARIA‐H in patients treated with anti‐amyloid mAbs, including lecanemab.^23^ Biologically, those who are ApoE ε4 carriers have a higher parenchymal and vascular Aβ load which predisposes the patient to a larger clearance of Aβ due to therapeutic effect, increased risk of transient cerebral amyloid angiopathy (CAA) and greater vascular permeability. Consequently, these factors predispose ApoE ε4 carriers to events of ARIA‐E and ARIA‐H.^25^, ^28^

It is estimated that approximately 15% of patients with AD are ApoE ε4 homozygous.^21^ The UK's MHRA Commission on Human Medicines (CHM) has advised that the benefits of lecanemab do not outweigh the risks in patients who are homozygous for ApoE4; however, the benefits outweigh the risks of treatment in patients who are heterozygous or are non‐carriers.^29^ This unfavourable benefit–risk balance in those with homozygous ApoE ε4 genotype status formed the basis of the re‐examination and subsequent approval of lecanemab by the EMA in November 2024. In the resubmission of data by the MAH, it was proposed to restrict the indication for lecanemab to patients with only one or no copy of ApoE ε4.^5^ As such, healthcare providers are required to screen patients for ApoE ε4 before treatment initiation and to not prescribe lecanemab to patients who are ApoE ε4 homozygotes.^21^

As part of the consideration of risks identified during the Clarity AD trial, it is important to note the strict exclusion criteria applied during the study to understand and predict possible risks that may be identified during routine clinical use. For example, whilst the inclusion of patients with baseline use of antithrombotic drugs, antiplatelet agents and anticoagulants was permitted in the Phase 3 clinical trial, the actual exposure to antithrombotic drugs (other than acetylsalicylic acid) was limited in the study cohort. Patients with other risk factors for intracerebral haemorrhage, including pre‐treatment MRI findings of prior cerebral haemorrhage, more than four microhaemorrhages, vasogenic oedema or superficial siderosis (findings suggestive of cerebral amyloid angiopathy) were also excluded from clinical trials.^30^

## 4 WHY IS THE CLINICAL TRIAL DATA LIMITED?

Pre‐marketing clinical trials are designed to assess the efficacy of an intervention of interest; however, these are often not designed to assess safety and are underpowered to detect AEs, particularly rare events. Strict recruitment criteria exclude patients at risk of certain AEs or comorbidities that, outside of a clinical trial setting, may influence the safety of the medicine.^31^ For example, patients were excluded if they had various risk factors for intracerebral haemorrhage and clinical trial data is limited for patients with exposure to antiplatelet agents (other than acetylsalicylic acid) or anticoagulants.^21^, ^30^ As such, the MHRA's CHM and the US FDA have advised that lecanemab is contraindicated for use in patients on blood‐thinning agents or who have cerebral amyloid angiopathy.^13^, ^14^ Additionally, NICE noted that people with young‐onset dementia, of diverse ethnicity and racial background, and a high lifetime risk of AD (such as people with Down's syndrome) were not sufficiently represented in the clinical trials.^3^ In addition, adherence to treatment in a real‐world setting is likely to differ compared with patients in clinical trials. For example, some patients may miss the fortnightly infusion because of holidays, transport problems or minor illnesses, resulting in a potential lowering of beneficial effect in routine use as compared to clinical trials. Because of these limitations, the generation of real‐world data regarding both the safety and effectiveness of this novel drug is crucial, particularly as lecanemab may have different benefits for different subgroups based on age, sex, ethnicity and adherence to therapy.^3^ Additionally, as the clinical trials do not include safety outcomes as a primary outcome, there is a limited understanding of the benefit–risk profile of this drug at present.^31^

When appraising the effectiveness and safety outcomes from the Phase 3 Clarity AD clinical trial, the limited duration of exposure of up to 18 months precludes a comprehensive understanding of the long‐term effectiveness of lecanemab and the detection of delayed SAEs.^32^ At present, this study has been extended to September 2027, with the primary outcome measures including the evaluation of the long‐term effects of lecanemab (as measured by a change from CDR‐SB score at baseline up to 69 months) and the number of participants reporting one or more treatment‐emergent adverse events (TEAEs) from the first dose of the study up to approximately 51 months (and 3 months follow‐up). This study extension includes participants from the core study unless the participants discontinued early. As of 9 July 2024, this included 1906 participants.^33^

## 5 THE NEED FOR A PHARMACOVIGILANCE PERSPECTIVE

The monitoring of the safety and effectiveness of lecanemab in real‐world clinical settings is required due to the acknowledged limitations of clinical trial data, including a restricted patient population, a sample size powered to detect efficacy outcomes and a limited duration of exposure.^31^ Following authorization of the drug across multiple countries/regions, the number of patients exposed to lecanemab will increase beyond the number of patients studied in clinical trials and will include a more diverse cohort of patients. There is therefore a reasonable expectation that new AEs, including SAEs, will be reported in the post‐authorization phase of the drug. Furthermore, there may be reports of more severe AEs previously observed in clinical trials, with potentially serious and life‐threatening events.

The post‐authorization monitoring of the complex benefit–risk profile of lecanemab in real‐world clinical use should encompass a broad range of pharmacovigilance activities. Post‐marketing surveillance of safety and effectiveness should inform a robust, ongoing review and assessment of the benefit–risk profile of lecanemab using a variety of available real‐world data (RWD) sources, with an understanding of the strengths and limitations of each data source used (Table 2).

**TABLE 2:** RWD sources applicable to the post‐marketing monitoring of lecanemab^1^, ^2^, ^34^.

Spontaneous reporting data		
Data sources	Strengths	Limitations
Individual case safety reports (ICSRs) of ADRs reported to: National and/or regional databases Global spontaneous reporting database	ADRs may be reported by both clinicians and patients. Used across clinical settings for all licensed and unlicensed indications (off‐label use). Electronic databases with large population coverage exist allowing advanced statistical techniques.	Prone to underreporting. Reports do not contain detailed descriptions of the exposure, diagnoses or other confounding factors. Varied completeness and quality of reports. Lack of control information, i.e. no available denominator data

Electronic health record data		
Data sources	Strengths	Limitations
Specialist care records Hospital inpatient and/or outpatient records Primary care medical records Emergency care records Dispensing records	Data reflects episodic care as captured within a specific institution or at a level of care. May include structured and unstructured data, including notes or other non‐standardized information.	Data across databases may have a non‐standardized data format. The level of completeness of data for each patient may vary between databases. Data may reflect the healthcare encounter only (and not the adherence of a patient to therapy).

Administrative data		
Data sources	Strengths	Limitations
Healthcare claims databases Procedures reimbursement	A comprehensive database of open (in‐process) and/or closed claims relating to patients covered. Claim and reimbursement records are often closely audited and/or validated.	Not inclusive of detailed clinical data or potential confounding variables. Exclude treatment or procedures accessed outside of the health plan/insurance system. Health plans have limited generalizability to the greater population.

Observational cohort data		
Data sources	Strengths	Limitations
Non‐interventional cohort studies Post‐authorization safety studies (PASS) Post‐authorization effectiveness studies (PAES)	Provides data such as incidence rates for an outcome of interest or multiple outcomes of interest for an exposure. Can include RWD collected through primary data collection and/or through secondary analysis of electronic healthcare databases for large cohorts.	Observational methods are more susceptible to bias and confounding. Require a large sample size and possibly a long study duration to detect rare outcomes.

Disease/condition‐specific registry data		
Data sources	Strengths	Limitations
Disease/condition‐specific registers ○ Dementia and/or AD registries ○ Death registries Drug registry data ○ AD and/or dementia drug registries	Collect specific patient data for groups with a certain disease, in a specific population, or on a specific drug of interest. A detailed source of data reflective of routine clinical practice that could be linked to electronic healthcare records. Reduced loss to follow‐up for the reference population.	Patient selection processes for registry enrolment could limit the representativeness of findings to the greater population. Data collected may only reflect the primary study objectives, limiting its applicability to secondary analysis.

Patient data		
Data sources	Strengths	Limitations
Patient‐reported outcomes (PROs) ○ Health surveys or questionnaires (carer and/or patients) ○ Interviews and/or focus groups (carer and/or patients) Patient‐generated health data ○ Wearable medical/personal devices ○ Internet‐based tools	Data from PROs is useful to inform clinical outcome assessments where a metric scale is unsuitable. Qualitative data from patient voice can be integrated to strengthen other sources of data. Technological advances facilitate the connection of researchers to patients for the direct collection of patient data.	Any data collected should be subject to the same fit‐for‐purpose assessments as other data sources. It may be challenging to harmonize data across different types of devices and/or wearables. Depending on how novel the data source is, more validation testing may be required.

Healthcare professional data		
Data sources	Strengths	Limitations
Clinician‐reported outcomes (ClinRO) ○ Prescriber surveys and/or questionnaires	Data may be used to inform clinical outcome assessments where patients may not be able to directly evaluate symptoms and functioning in their daily life. Are useful when data is required in which biomarkers alone are not representative of the outcome of interest.	Data derived is reliant on a clinician's interpretation of an outcome, introducing bias. The ability of clinicians to engage with surveys is likely to be less in the real world compared to more controlled settings of clinical trials.

1. The European Network of Centres for Pharmacoepidemiology and Pharmacovigilance (ENCePP). Guide on Methodological Standards in Pharmacoepidemiology (Revision 11). 2010. EMA/95098/2010.

2. Powers JH, Patrick DL, Walton MK, Marquis P, Cano S, Hobart J, et al. Clinician‐reported outcome assessments of treatment benefit: report of the ISPOR Clinical Outcome Assessment Emerging Good Practices Task Force. *Value in Health*. 2017;20(1):2‐14.

3. Strom BL. Choosing among the available data sources for pharmacoepidemiology research. In: Strom BL, Kimmel SE, Hennessy S, eds. Pharmacoepidemiology. 6th ed. Wiley; 2019: 355–371.

As the cornerstone of pharmacovigilance, a proactive review of spontaneous reports of adverse drug reactions (ADRs) reported from healthcare professionals and patients across national and global databases should be conducted, involving the review of individual case safety reports (ICSRs). Spontaneous reporting systems facilitate the early detection of safety concerns following marketing approval, which can be country‐ or region‐specific. To align with regulatory requirements, associated signal detection and management activities should be ongoing to identify new AEs or changes to known AEs observed with lecanemab in the real world. The periodic review of confirmed safety signals should inform the ongoing benefit–risk assessment of the product throughout the product's lifecycle.^35^

Spontaneous reporting data should always be used in conjunction with other RWD sources due to its limitations, including underreporting, lack of a denominator or control data, as well as variable reporting for highly publicized drugs.^36^ Data regarding safety outcomes should be obtained from observational studies using a variety of RWD sources. The choice of the most appropriate data source(s) depends on the availability of the data of interest, for example, relating to utilization, safety and/or effectiveness. Note that it is likely that the utilization of lecanemab in the UK may be limited and delayed in the initial post‐authorization phase. This may impact the availability of sufficient RWD to detect rare AEs with suitable statistical power during this phase.^37^ It is also important to consider that RWD sources, such as electronic health records (EHR) and/or administrative databases, may also be representative of drug availability at a specific level‐of‐care or of particular patient demographics. For example, data for patients prescribed lecanemab in the UK's private sector will unlikely be recorded in electronic primary care databases such as the Clinical Practice Data Link (CPRD). Furthermore, the demographics of patients accessing lecanemab through the private sector will differ to those accessing healthcare services through the UK's NHS.

A post‐authorization safety study (PASS) has been requested as a condition of the marketing approval by regulators across global contexts, including the US, UK, Europe and Japan. The EMA require a PASS as a condition of its approval which is intended to utilize registry data with coverage in the European Union.^5^ In the US, a non‐interventional post‐marketing study is being conducted linked to the Alzheimer's Network for Treatment and Diagnostics (ALZ‐NET) registry collecting RWD for patients being evaluated for memory concerns or receiving novel FDA‐approved treatments for AD.^38^ In the case of gantenerumab, an anti‐amyloid mAb currently in clinical trials, the feasibility of linking clinical trial data from the GRADUATE Phase 3 trial with real‐world claims data across 21 sites across the US has been explored.^39^ These studies demonstrate the power of linking data between RWD sources to strengthen evidence generation for novel AD drugs in the real‐world setting.

The use of lecanemab in the clinical setting should be accompanied by RMMs to optimize the risk–benefit balance of the product. These include baseline diagnostic tests to confirm the presence of Aβ pathology using either a positron emission tomography (PET) scan, cerebrospinal fluid (CSF) analysis or equivalent validated methods. In addition, it is recommended that the patient has a baseline MRI scan (to identify any pre‐existing ARIA or brain pathology) with further MRI scans to identify AEs, particularly ARIA, prior to the 5th, 7th and 14th infusions.^21^, ^30^ Pharmacogenomic testing is recommended as there has been an increased incidence of ARIA in patients who are ApoE ε4 homozygotes compared to patients who are heterozygotes and non‐carriers in clinical trials.^1^, ^14^, ^15^ Clinical vigilance of all patients for ARIA during the first 14 weeks of treatment with lecanemab is necessary, with additional MRIs if symptoms are experienced. Dosage interruptions may be required based on observed ARIA‐E or ARIA‐H and severity of MRI, with interruption and resumption of therapy determined by clinical judgement.^21^, ^30^ Importantly, the UK's MHRA has indicated that the initiation of treatment of all patients will be through a central registration system as part of the controlled access programme as an additional RMM. Furthermore, a PASS may be undertaken to generate real‐world data on the safety and benefit–risk profile of lecanemab.^8^

## 6 MANAGING THE RISKS IN CLINICAL PRACTICE

Informed by the risks identified in clinical trials, pharmacovigilance planning is required throughout a drug's lifecycle and involves activities required to further characterize and minimize associated risks. The RMMs for a drug should be proportionate to the identified and potential risks associated with the authorized indication and the need to generate post‐authorization data.^40^ As a key component of pharmacovigilance, routine RMMs should be implemented for the safe use of a drug to protect the health of the public across various settings.^41^

Concerns have been raised regarding the feasibility of conducting the proposed RMMs to manage the risks of lecanemab in real‐world clinical settings.^42^ The proposed RMMs include conducting PET and MRI scans at baseline and repeated MRI scans, in addition to the required pharmacogenomic testing (ApoE ε4) prior to initiating treatment. These RMMs for lecanemab require significant patient compliance and are resource‐ and labour‐intensive. For example, patient compliance with extensive scanning schedules may be difficult to achieve due to behaviours associated with early AD, such as confusion and difficulty concentrating. Furthermore, timely and frequent access to MRI scanning facilities (both routinely and if events of symptomatic ARIA are suspected) and sophisticated genotype testing are resource‐intensive and require suitably experienced and trained health personnel.^42^ It is unclear to what extent the implementation of these RMMs in low‐resource settings and/or in overburdened health systems are feasible, across both low‐ and middle‐income countries (LMICs) and high‐income countries (HICs) alike. It is important that the evaluation of the effectiveness of these RMMs complements the use of lecanemab in real‐world settings. This includes an assessment of the knowledge of the risks (communicated through the provision of a healthcare professional guide and a patient alert card), changes in behaviours (such as adherence to scanning schedules) and the evaluation of rates of AEs.

For example, NICE in the UK has noted that the use of lecanemab as part of routine care for patients with early AD would require a significant increase in NHS capacity. The routine use of lecanemab in the NHS would disrupt current treatment pathways for the disease and substantially increase the demand for supportive services in primary care and memory clinics. Treatment with lecanemab would comprise twice weekly intravenous (IV) infusions initiated in secondary care. Thereafter, routine outpatient follow‐up appointments would be required every 3 months with a requirement for routine MRIs during treatment, including the need for any acute management of ARIA, if detected, involving additional MRI. Currently, NICE has considered the high cost of the drug itself (and the costs of infusion and associated clinical care) an unfavourable use of NHS resources.^3^

Additionally, the routine use of lecanemab would lead to the requirement of a new diagnostic pathway in the NHS. In particular, this would include the set‐up of specialist diagnostic clinics to provide confirmatory diagnostic tests by lumbar puncture or a PET‐CT scan, and pharmacogenetic testing for ApoE ε4.^3^ Similar challenges exist in the US as although it is required that pharmacogenetic testing for ApoE ε4 status is conducted prior to treatment, US product information cautions that an FDA‐authorized test for the detection of ApoE ε4 alleles is not currently available with current tests varying in accuracy and design.^21^ As the presence of an ApoE ε4 allele increases the risk of ARIA, CAA and intracerebral haemorrhage, these diagnostic limitations in the real‐world clinical setting raise concerns over the suitability of the RMMs to healthcare systems globally. Such limitations may be more apparent in fragmented healthcare systems in LMICs where weak regulatory oversight and poor enforcement of legislation is more common.^43^

## 7 CONCLUSION

Despite the identified risks, the development of anti‐amyloid mAbs, such as lecanemab, provides a potential novel disease‐modifying treatment pathway and represents a significant step forward in AD research. With numerous anti‐amyloid mAbs in various stages of drug development, understanding the benefits and risks of these drugs is an essential component of their safe and effective use.^44^ In the UK, the MHRA's decision to grant a marketing approval for lecanemab, followed by the non‐recommendation by the UK's NICE, highlights the existing uncertainties around the ongoing benefit–risk profile of the product and its cost‐effectiveness. Concerns regarding the capacities of healthcare systems to deliver anti‐amyloid mAbs safely highlights the crucial need to monitor how effective RMMs are implemented across real‐world settings. As increased RWD emerges across various clinical settings, comprehensive assessment of safety evidence is crucial to protect the health of patients with AD.

### 7.1 Nomenclature of targets and ligands

Key protein targets and ligands in this article are hyperlinked to corresponding entries in http://www.guidetopharmacology.org, and are permanently archived in the IUPHAR/BPS Guide to PHARMACOLOGY.^34^

## AUTHOR CONTRIBUTIONS

A.B. was responsible for sourcing policy/literature, interpretation, developing diagrams and figures, and drafting and finalizing the manuscript. M.D., E.L., D.R. and A.Y. critically reviewed and supported the interpretation of data. S.A.W.S. was responsible for the study conception, interpretation and critical review of the manuscript. All authors approved the final version of the manuscript for publication.

## CONFLICT OF INTEREST STATEMENT

None of the authors disclose any conflict of interest directly relevant or directly related to this work.

## Data Availability

Data sharing is not applicable to this article as no datasets were generated or analysed.
